# Acromegaly: Pathophysiological Considerations and Treatment Options Including the Evolving Role of Oral Somatostatin Analogs

**DOI:** 10.3390/pathophysiology30030029

**Published:** 2023-09-01

**Authors:** Charles P. Daniel, Maxwell J. Wagner, Grant E. Borne, Connor J. Plaisance, Shahab Ahmadzadeh, Alfonso Aquino, Sahar Shekoohi, Adam M. Kaye, Elyse M. Cornett, Alan D. Kaye

**Affiliations:** 1 School of Medicine, Louisiana State University Health Sciences Center at Shreveport, 1501 Kings Highway, Shreveport, LA 71103, USA; cpd002@lsuhs.edu (C.P.D.); mjw002@lsuhs.edu (M.J.W.); geb002@lsuhs.edu (G.E.B.); cjp001@lsuhs.edu (C.J.P.); 2 Department of Anesthesiology, Louisiana State University Health Sciences Center at Shreveport, 1501 Kings Highway, Shreveport, LA 71103, USA; shahab.ahmadzadeh@lsuhs.edu (S.A.); alfonso.aquino@lsuhs.edu (A.A.); elyse.bradley@lsuhs.edu (E.M.C.); 3 Department of Pharmacy Practice, Thomas J. Long School of Pharmacy and Health Sciences, University of the Pacific, Stockton, CA 95211, USA; akaye@pacific.edu; 4 Departments of Anesthesiology and Pharmacology, Toxicology, and Neurosciences, Louisiana State University Health Sciences Center at Shreveport, 1501 Kings Highway, Shreveport, LA 71103, USA; alan.kaye@lsuhs.edu

**Keywords:** acromegaly, Mycapssa^®^ (oral octreatide), somatostatin analogs, growth hormone, IGF-1

## Abstract

Acromegaly is a condition most commonly diagnosed in the fifth decade of life and has numerous treatment options. In this regard, Mycapssa^®^ is the first FDA-approved oral octreotide capsule for treating acromegaly, combining the efficacy of the somatostatin receptor ligand, octreotide, with the ease of a twice-daily oral capsule. Where surgical treatment is not an option, somatostatin analogs, including octreotide, are the first line of medical treatment for acromegaly, requiring regular subcutaneous or intramuscular injections administered by a patient’s healthcare provider. Octreotide capsules (Mycapssa^®^) provide an alternative to these somatostatin receptor ligand injections by combining octreotide with other excipients to produce a transient permeability enhancer technology that improves paracellular transport of octreotide across the gastrointestinal wall into the small intestine. Across multiple trials, including open-label (CH-ACM-01), double-blind placebo-controlled (CHIASMA OPTIMAL), and open-label extension of the trial period (CHIASMA OPTIMAL OLE), Mycapssa^®^ octreotide capsules maintained a consistent biochemical normalization of IGF-1 and GH levels, safety profiles similar to injected somatostatin receptor ligands, and patient preference to continued treatment with octreotide capsules. While clinical trial data supports the use of octreotide capsules (Mycapssa^®^) in the pharmacological management of GH and IGF-1 levels, very little data exist regarding the drug’s efficacy, tolerability, and use in female or pediatric-specific populations. A better understanding of the efficacy, application, and role of oral octreotide capsules in the long-term medical management of acromegaly in a diversity of populations is imperative to best determine the risks/benefits for the clinician.

## 1. Introduction

Acromegaly is a disease of excessive growth hormone production by the anterior pituitary gland, resulting in dysfunction in metabolic and physical development. Patients with acromegaly tend to have chronically elevated GH and IGF-1 levels, which cause excessive growth of body tissues characterized by the clinical findings of a prominent forehead, large jaw, large hands and feet, and thickened skin [1,2,3]. The systemic effects of acromegaly, including cardiovascular, respiratory, metabolic, and neurologic complications, lead to elevated mortality in these patients, which should be managed by suppressing GH and IGF-1 levels to that of the normal population [4]. Medical management of these patients primarily indicates surgical resection of the pituitary adenoma or medical therapy. Of the medical therapy options, the somatostatin analog class of drugs is the first line of medical therapy for acromegaly with clinical efficacy in managing GH levels, IGF-1 levels, and suppression of tumor growth. Octreotide is a somatostatin receptor ligand from this class of drugs commonly used in medical therapy for acromegaly. While effective, somatostatin receptor ligands require regular subcutaneous or intramuscular injections which risks injection site pain and adverse effects as well as requiring most patients to regularly visit their medical provider for these injections in long-term disease management [5].

In an attempt to combat the administrative strain upon patients and healthcare providers, oral delivery of the somatostatin receptor ligand, octreotide, eliminates the risks and challenges of regular injections. As the only FDA-approved oral octreotide capsule for long-term medical treatment of acromegaly, the Mycapssa^®^ twice-daily oral capsule suggests a new opportunity for at-home long-term management of GH and IGF-1 levels in patients with acromegaly [5,6].

Few studies have analyzed the spectrum of available treatments for managing GH and IGF-1 levels in acromegaly, focusing on the efficacy, safety, and patient satisfaction of treatment with octreotide capsules (Mycapssa^®^) across multiple clinical trials and open-label extensions of these trials. Likewise, few studies have assessed the acromegaly disease burden, challenges, and pharmaceutical management within female and pediatric populations with an emphasis on drug safety, tolerability, and risks within these unique populations. The present investigation, therefore, searches to assess current treatment options and investigate applications, safety, and efficacy of oral octreotide capsules in a diversity of populations to explore where octreotide capsules (Mycapssa^®^) may aid in reducing both patient and medical provider strain by providing long-term, at-home management of acromegaly.

## 2. Acromegaly: Review of Disease Pathology

### 2.1. Acromegaly Etiology

Acromegaly is a disorder of the anterior pituitary which results in the overproduction of growth hormone (GH). The hypothalamic peptide growth hormone-releasing hormone (GHRH) provides the primary stimulus for the production of GH, while somatostatin provides negative feedback, decreasing GH secretion. GHRH secretion is modulated by a variety of factors, including sleep, diet, stress, and exercise. Somatostatin secretion is stimulated by factors including increased IGF-1 levels and low blood glucose levels. Excess GH also provides negative feedback to decrease pituitary GH secretion when serum GH levels are elevated [4,7]. Pituitary GH secretion stimulates the production and release of insulin-like growth factor 1, IGF-1, from the liver. This subsequent disorder of hypersecretion of these stimulatory growth hormones, acromegaly, manifests as a product of the synergistic effects of tissue overgrowth produced by GH and IGF-1. Acromegaly is commonly identified by these systemic clinical effects of GH excess, including metabolic and physical development dysfunction [4,8]. There are three primary etiologies by which this disease of GH excess manifests: primary GH excess, growth hormone-releasing hormone (GHRH) excess, and ectopic/iatrogenic GH excess. Of these three etiologies, primary GH excess is the most prominent subtype, manifesting as an adenoma of the anterior pituitary gland that secretes GH [2]. Other notable causes within the class of primary GH excess include GH cell carcinomas and multi-hormone-producing pituitary adenomas [9,10]. The second most prevalent etiology, ectopic/iatrogenic GH excess, is caused by either excessive GH administration or production by not-pituitary tumors like lymphomas and pancreatic-islet cell tumors. The last etiology of GH excess, GHRH excess, includes both central and peripheral mechanisms of excess production of the GHRH, which serves as a stimulatory hormone for pituitary GH release. The most prevalent central causes of GHRH excess include hypothalamic hamartomas, ganglioneuromas, and choristoma. Peripheral mechanisms of GHRH secretion excess include bronchial carcinoid tumors, adrenal adenomas, small-cell lung cancer, and pheochromocytomas [4,8].

### 2.2. Acromegaly Clinical Diagnosis

Acromegaly is a disorder with a slow progression, with clinical presentation often going undiagnosed until the third decade of life, with the mean age of diagnosis at 40 in men and 45 in women. Women account for 49.6% of the new diagnoses of acromegaly, exhibiting a nearly balanced gender distribution of disease prevalence [11]. Screening for acromegaly is typically prompted by a patient with the characteristic phenotypic features of acromegaly or a presentation with multiple diagnoses with associated disorders. The phenotypic features of acromegaly include an enlarged lower jaw, large hands and feet, a prominent forehead, deepening of the voice, hypertrophic arthropathy, coarse facial features, and many other physical characteristics due to the systemic effects of GH excess. While the diagnostic criteria for acromegaly are identical in both male and female populations, females experience an average delay in diagnosis of 3.1 years when compared to their male counterparts [11]. Commonly associated disorders that may suggest screening for acromegaly if multiply present in a single patient include sleep apnea, hypertension, debilitating arthropathy, type 2 diabetes mellitus, and carpel tunnel syndrome [4,8,12].

Screening for acromegaly is primarily accomplished through the biochemical evaluation of IGF-1 due to the highly variable daily fluctuations of GH concentration related to temporal factors, exercise, and sleep patterns. An elevated IGF-1 level is diagnostic of GH excess, while a normal IGF-1 level effectively rules out the possibility of acromegaly. If a patient presents with an ambiguous IGF-1 level, but physiologic or metabolic clinical findings still suggest acromegaly, a GH suppression test can be performed to measure GH levels before and two hours after a 75 g glucose load is orally administered. A diagnosis of acromegaly is confirmed by GH concentrations > 1 ng/mL two hours after the glucose load. If IGF-1 is elevated or the GH suppression test is positive, these findings should prompt imaging studies to localize the GH-secreting tumor [4,8].

### 2.3. Acromegaly Prognosis

The prognosis of acromegaly largely depends on the response to disease management and treatment. The mortality rate of individuals with acromegaly typically ranges from 1.2 to 3.3 times that of the general population, with the primary contributing factors including malignancies, cardiovascular complications, and respiratory disorders. These mortality-contributing complications include sleep apnea, insulin resistance, diabetes, hypertension, arrhythmias, carpel tunnel, skin thickening, cartilage hypertrophy, and periosteal bone formation [2]. Gender is also associated with acromegaly disease prognosis and quality of life in acromegaly due to a reported average diagnostic delay of 3.1 years in females, leading to an increased prevalence of disease-associated complications, including hypertension and diabetes within this population [13]. The key predictor of the case-by-case mortality rate in acromegaly is the post-operative GH levels, with controlled GH/IGF-1 levels increasing life expectancy to that of the age-matched controls. The presence of high GH/IGF levels, hypertension, and cardiomyopathy contribute to a worse prognosis. The prognosis of acromegaly is classified into three subtypes: prognosis, treatment responsiveness, and patient outcome. Type 1 signifies the best prognosis, type 2 signifies an intermediate prognosis, and type 3 suggests the worst prognosis. The primary factors that define this system of classification and prognosis are age at diagnosis (young suggesting higher numerical grade), GH/IGF-1 levels (high suggesting higher numerical grade), tumor granularity (sparse granularity suggesting higher numerical grade), invasion (invasive features suggesting higher numerical grade), and size (larger tumors suggesting higher numerical grade), specific mutations, receptor expression (low SSTR2 expression suggesting higher numerical grade), and markers (low p21 levels suggesting higher numerical grade) [4]. Normalization of both IGF-1 and GH is the primary aim of therapies targeted to treat acromegaly due to the well-supported improvement in patient outcomes and decreased mortality when normalization of hormone levels is achieved [14,15]. Given the attenuation of morbidity and mortality with disease management and a global presence of about 116.9 new cases per million people diagnosed each year, appropriate screening, early diagnosis, and patient education are necessary to achieve the best outcomes in patients with this disorder [4,8].

## 3. Acromegaly Treatment

### 3.1. Goals in Treatment of Acromegaly

Treatment of acromegaly focuses primarily on control of the GH and IGF-1 levels to improve the patient’s prognosis while also managing the impact of associated symptoms on the patient’s quality of life. Treatment for acromegaly can be subdivided into two approaches: surgical and medical therapy. The surgical approach prevails in treating pituitary adenomas due to this modality’s relief of mass effect-associated symptoms and improvement of the subsequent response to medical treatment when the surgery is non-curative. Medical therapy for acromegaly is considered for patients who may not be good surgical candidates, as neoadjuvant therapy before surgery, and for those with recurrent disease after initial tumor resection but do not qualify for repeat surgery. Somatostatin analogs remain the first line of treatment because of their clinical efficacy in suppressing the secretion of GH, IGF-1, and suppression of tumor growth. Other drug classes are incapable of this spectrum of biological management of acromegaly [2,4,14].

### 3.2. Surgical Treatment of Acromegaly

The best predictors of surgical success in acromegaly are smaller tumor size, absence of invasion into surrounding structures, and lower GH and IGF-1. The three most prevalent surgical approaches to GH-secreting pituitary adenomas are endoscopic transsphenoidal surgery, transsphenoidal microscopic surgery, and craniotomy. Endoscopic transsphenoidal surgery utilizes an endoscope inserted through a small incision in the nose or upper lip, providing the best tumor visualization of the three surgical approaches. The second approach, transsphenoidal microscopic surgery, is the traditional method of transsphenoidal surgery, which directly visualizes the tumor with a microscope. The endoscopic and microscopic approaches demonstrate comparable reemission rates; however, the endoscopic approach theoretically offers improved panoramic visualization of the tumor, which the microscopic approach cannot. The third surgical approach, craniotomy, is reserved for invasive adenomas with suprasellar extension and cases with para-stellar sinuses and carotid artery aneurysms. Post-surgical management includes immediate post-surgical monitoring of urine sodium output, adrenal function, and GH secretion. Post-surgical GH and IGF-1 measurements are done at one week, three months, six months, and annually due to the risk of relapse after confirmed remission. Post-surgical imaging should also be done at three months post-operation to allow adequate gel and foam packing resorption time. If residual disease is present, options include repeat surgery, medical management, and radiotherapy [2,4].

### 3.3. Medical and Radiotherapy Treatment of Acromegaly

Medical therapy is appropriate in managing acromegaly where surgery is no longer an option due to patient desires, unresectable tumors, high surgical risks, and post-operative disease recurrence. The three most prevalent medical therapy approaches to acromegaly are somatostatin analogs, dopamine receptor agonists, and GH receptor antagonists (see Table 1). The first drug class, somatostatin analogs, the first line of medical therapy for acromegaly, is a class of drugs that are a synthetic form of somatostatin. These drugs bind to somatostatin receptors found on both the normal pituitary gland and the somatotroph adenoma to suppress GH secretion by both the normal pituitary and the tumor. Somatostatin analogs also reduce GH binding to hepatocytes, decreasing IGF-1 production. Administration of somatostatin analogs typically follows a schedule of once-monthly intramuscular injections or subcutaneous injections 3–4 times daily. The second drug class, dopamine receptor agonists, acts upon the pituitary gland dopamine 2 receptors (D2R) to suppress GH secretion. Dopamine receptor agonists are less effective than somatostatin analogs at hormonal control and tumor size reduction. They, therefore, are typically only indicated as adjunctive therapy with somatostatin analogs or in mild disease presentations. The third drug class, GH receptor antagonists, is a class of human growth hormone analogs that lower IGF-1 levels through blockage of the GH receptors from endogenous GH. GH receptor antagonists are unable to suppress endogenous GH production. Therefore, GH levels may be elevated while IGF-1 levels are suppressed, rendering IGF-1 levels the only effective treatment monitor. Another limitation of GH receptor antagonists is that they lack anti-proliferative effects and will not reduce tumor size. Somatostatin analogs remain the primary medical therapy for acromegaly, with dopamine receptor agonists and GH receptor agonists serving as alternative or adjunctive therapies for patients with poor tolerance or resistance to somatostatin analogs. Lastly, radiotherapy is reserved for patients with post-surgical disease recurrence and failure of medical therapy in disease control. The two suggested approaches for radiotherapy are conventionally fractionated radiotherapy and stereotactic radiotherapy; both pose the risk of hypopituitarism and irradiation damage to adjacent brain tissue [4].

### 3.4. Somatostatin Analogs in Acromegaly Treatment

The first-line pharmaceutical approach to the treatment of acromegaly is the somatostatin analog class of drugs which mimic the physiological actions of somatostatin. The cyclic polypeptide somatostatin acts as one of the primary inhibitors of endocrine and exocrine hormone secretion with suppressive effects on a variety of hormones, including GH and anti-proliferative effects, reversing the impact of IGF-1’s mitogenic signals. Native somatostatin is extremely clinically limited due to its 1–3 min half-life and rapid degradation by peptidases in plasma and tissues. Somatostatin induces its biological effects via a subset of G protein couple receptors (SSTR1-SSTR5) [16].

To overcome the limited bioavailability of native somatostatin, synthetic somatostatin analogs were developed, with three gaining FDA approval: octreotide, lanreotide, and pasireotide. The first group of synthetic somatostatin analogs, octapeptide octreotide (Sandostatin^®^), gained FDA approval in 1988. The octapeptide octreotide (Sandostatin^®^) showed high affinity for SSTR2 and required subcutaneous dosing of 50, 100, or 200 mg every 8–12 h. Later, in 1998, the first long-acting release synthetic somatostatin analog (Sandostatin LAR^®^) gained FDA approval. The long-acting release formulation (Sandostatin LAR^®^) contained octreotide within polymer microspheres with an intramuscular dosing regiment at doses of 10, 20, or 30 mg every 28 days. The third formulation, octreotide capsules (Mycapssa^®^), gained FDA approval in 2020. The oral octreotide capsules (Mycapssa^®^) are orally administered as twice daily 20 mg capsules with a maximum dosage recommendation of 80 mg daily. The second group of synthetic somatostatin analogs, the cyclic octapeptide lanreotide, was developed in the 1990s with an initial half-life of only 90 min. Later, a sustained-release formulation of lanreotide (Lanreotide SR) was developed with a half-life of 4.5 days. The lanreotide sustained release formulation required intramuscular injection with 30 or 60 mg dosage every 7–14 days. Lanreotide was then further developed into a sustained release aqueous formation (Autogrel^®^) requiring a subcutaneous dosing regimen of 60, 90, or 120 mg every 28 days. Lanreotide (Somatuline^®^) gained FDA approval in the United States for acromegaly treatment in 2007. The third group of synthetic somatostatin analogs, pasireotide, binds with higher affinity than octreotide and lanreotide to the somatostatin receptors SSTR1, SSTR3, and SSTR5. Pasreotide LAR (Signifor LAR^®^) gained FDA approval in 2014, with an intramuscular dosing regimen of 20, 40, or 60 mg every 28 days [16,17].

GH-secreting pituitary adenomas (somatotropinomas), seen commonly in acromegaly, predominantly express SSTR2, with the second most common somatostatin receptor expressed being SSTR5. Higher SSTR2 expression is correlated to improved response to somatostatin analog treatment, while low SSTR2 expression allows resistance. All three groups of somatostatin analogs, octreotide, lanreotide, and pasireotide, show an identical affinity for the SSTR2 receptor, with pasireotide showing superior affinity to the SSTR5 receptor when compared to other somatostatin analog groups [16].

### 3.5. Treatment Considerations in Women

Acromegaly shows a relatively balanced gender distribution, with research suggesting 49.6% of new diagnoses of acromegaly cases present in females. Females tend to be older, with a mean age difference of 3.1 years compared to males at diagnosis. Female acromegaly also presents with lower IGF-1 levels and comparably identical GH levels and pituitary adenoma size to the male population [11]. It has been shown that with the diagnostic delay and higher GH relative to the IGF-1 ratio seen in female acromegaly, women experience a more adversely affected quality of life with hypertension and diabetes. Surgical outcomes in female acromegaly management are identical to the male population’s. Pharmaceutical management of both male and female patients with acromegaly recommends equal responsiveness to somatostatin analogs, including octreotide. However, delayed diagnosis in females increases the disease burden, often requiring higher dosages. With the normalization of GH and IGF-1 levels, the mortality rate is comparable to that of the general population [13].

### 3.6. Treatment Considerations in Pediatrics

Within the pediatric population, the pathology of GH hypersecretion most commonly manifests under gigantism. The defining difference between a diagnosis of acromegaly and gigantism is that gigantism is identified by GH hypersecretion before epiphyseal fusion within the growth plates of long bones. Gigantism affects pediatric patients during infancy, childhood, and adolescence, presenting with tall stature (3 standard deviations above the mean height) alongside other clinically diagnostic presentations of acromegaly [8]. While pediatric gigantism can manifest as a sporadic pituitary tumor leading to GH excess, it is also highly related to genetic predisposition and various syndromic diseases, including neurofibromatosis, McCune Albright syndrome, and Carney complex, therefore making the medical management rather challenging [18]. Due to the limitations of large-scale studies evaluating various therapeutic approaches to GH excess in pediatric patients, optimal treatment of gigantism is typically extrapolated from adult literature and follows the modalities of surgery, radiation, and medical therapy. Of the pharmacologic approaches, somatostatin analogs, including octreotide, have been used safely in children with gigantism for long-term management and normalization of GH and IGF-1 levels both alone and when combined with dopamine analogs [19].

## 4. Mycapssa^®^: Oral Octreotide Capsule

### 4.1. Oral Octreotide Capsule vs. Subcutaneous Somatostatin Receptor Ligands

Mycapssa^®^, an oral octreotide capsule for treating acromegaly, received FDA approval in 2020 and provides an alternative method of drug administration that may prove more convenient than the standard subcutaneous or intramuscular injections of octreotide [20]. Octreotide, a synthetic version of somatostatin, is a somatostatin analog drug class member. Somatostatin analogs have proven effective in suppressing GH secretion by the pituitary gland and pituitary adenomas, as well as efficacy in suppressing hepatocyte IGF-1 production and in arresting tumor growth. For these reasons, somatostatin analogs remain the far and away first-line medical treatment of acromegaly with support for treatment efficacy in patients before surgery, post-surgery, and in cases where surgery is not an option for treatment [4]. Traditionally, somatostatin analogs have been administered through monthly intramuscular injections or three to four daily subcutaneous injections. Intramuscular injections of somatostatin receptor analogs like octreotide and lanreotide are the current first-line medical treatment for acromegaly; however, these deep tissue injections may cause injection site pain, nodules, bruising, inflammation, and scarring. While some patients can administer these injections at home, roughly only 17% of patients with acromegaly can maintain treatment in this manner. Thus, most patients are required to see a healthcare provider every 28 days for intramuscular injections. Octreotide capsules (Mycapssa^®^) have revolutionized this industry by becoming the first FDA-approved oral therapy for the long-term treatment of acromegaly to address the limitations of medical treatment by delivering the somatostatin receptor ligand, octreotide, in a convenient oral capsule taken twice daily [5].

### 4.2. Oral Octreotide Capsule: Mechanism of Action

Somatostatin analogs are a class of acromegaly-treating drugs that bind to somatostatin receptors and suppress GH release from both the pituitary gland and the somatotroph adenomas found in acromegaly. Somatostatin analogs are also effective in suppressing IGF-1 production due to the suppression of GH secretion and subsequently decreased binding of GH to hepatocytes [2,4]. Mycapssa^®^ is an oral capsule formulation of octreotide, a synthetic octapeptide human somatostatin analog. Octreotide’s synthetic formulation has a half-life of approximately 100 min compared to 2–3 min for endogenous somatostatin in humans. Typically, octreotide exhibits low bioavailability with oral administration, therefore limiting treatment to the parenteral route. The greatest contributors to oral octreotide’s low bioavailability are its low enzymatic stability and poor membrane permeability within the lumen of the gastrointestinal tract [21,22]. Octreotide capsules (Mycapssa^®^) have overcome these limitations with a new formulation combining octreotide with other excipients to produce a transient permeation enhancer (TPE) technology. The TPE technology is produced by octreotide’s combination with excipients, yielding an oily suspension of hydrophilic particles in a lipophilic medium. This suspension can inflict transcellular perturbation of the intestinal epithelial barrier, which causes transient opening of the intestinal epithelial tight junctions. This TPE technology has been shown to effectively facilitate the paracellular transport of octreotide across the gastrointestinal wall into the small intestine [20,21,23,24]. A study comparing subcutaneous octreotide with oral octreotide found that oral octreotide absorption into circulation was present within 1 h of treatment. The 20 mg oral dose of oral octreotide demonstrated an equivalent pharmacokinetic profile to that of 0.1 mg of subcutaneous octreotide when assessing peak plasma volume, rate of plasma decay, and mean area under the curve [21]. Octreotide capsules (Mycapssa^®^) have established clinical efficacy in maintaining normalization of GH and IGF-1 levels equivalent to those of parenteral octreotide injections in trials of both healthy patients and those with diagnosed acromegaly. In the open-label extension period of clinical trials, octreotide capsules (Mycapssa^®^) showed no increased susceptibility to adverse events and improved gastrointestinal tolerability when used for long-term acromegaly management. With demonstrated efficacy, safety, and ease of use for patients, the octreotide capsules (Mycapssa^®^) earned FDA approval in 2020 as the only approved oral octreotide capsule [5,20,21,25].

### 4.3. Oral Octreotide Capsule: Clinical Efficacy and Safety

Octreotide capsules (Mycapssa^®^) have undergone trials including open-label (CH-ACM-01), double-blind placebo-controlled (CHIASMA OPTIMAL), and open-label extension of the trial period (CHIASMA OPTIMAL OLE), all while maintaining a consistent biochemical response, safety profiles similar to injected somatostatin receptor ligands, and patient preference to continued treatment with oral octreotide capsules. The CH-ACM-01 trial enrolled 155 acromegaly patients who were previously responsive to treatment with injected somatostatin receptor ligands and found that oral octreotide capsules effectively maintained a biochemical response equivalent to injected somatostatin receptor ligands in 65% of patients [6]. The CHIASMA OPTIMAL trial enrolled 56 acromegaly patients previously responsive to treatment with injected somatostatin receptor ligands and found that 58.2% of patients maintained an equivalent biochemical IGF-1 level and 77.7% maintained an equivalent GH level to that of an injectable somatostatin receptor ligand [25]. Mean IGF-1 levels were maintained within the inclusion criteria throughout both trials. After these trials, over 85% of the treated patients elected to continue treatment with the oral octreotide capsules into the extension phases of the study. When considering oral octreotide capsules, the most frequently reported adverse events were gastrointestinal, neurological, and musculoskeletal, all consistent with the known subcutaneous octreotide capsules’ safety profile. Analysis of adverse events and safety profiles in both the CH-ACM-01 trial and the CHIASMA OPTIMAL trial suggests that the oral octreotide capsules safety profile is consistent with that of injected somatostatin receptor ligands and the acromegaly disease burden. No new risks or safety signals outside those consistent with injected octreotide were identified during the CHIASMA OPTIMAL or CH-ACM-01 trials [1,26]. A subsequent MPOWERED trial assessed the safety of oral octreotide capsules with that of injected somatostatin receptors through a 36-week randomized controlled treatment monitoring adverse events while treating one group with injected somatostatin receptor ligands and the other with oral octreotide capsules. The MPOWERED trial found that the oral octreotide capsules’ safety profile was consistent with that of the injected somatostatin receptor ligands [26]. Contraindications for the use of octreotide capsules (Mycapssa^®^) include hypersensitivity to octreotide or any of the components of Mycapssa^®^ due to reports of anaphylactoid reactions in those receiving octreotide [17]. Oral octreotide capsules were well tolerated throughout all clinical trials and the extension period. The most commonly reported adverse symptom was gastrointestinal discomfort, including flatulence, nausea, and diarrhea, which typically improved with long-term treatment (see Table 2) [6,26].

### 4.4. Oral Octreotide Capsule: Considerations in Women and Pediatrics

Within the female population, the use of octreotide capsules (Mycapssa^®^) should consider the risk of increased fertility and the suggested risk of the drug passing into breast milk. Within the premenopausal female population, octreotide’s therapeutic benefits of normalized GH and IGF-1 may lead to improved fertility, posing the risk of unintended pregnancy in this population. While no data have been shown to evidence the passing of octreotide into human breast milk, subsequent effects on the breastfed infant, or drug effects on lactation, animal studies have evidenced lactational octreotide transfer. Subcutaneous octreotide injections in lactating rats have resulted in the presence of octreotide in the milk of these rats, suggesting the drug might also be present in human breast milk. Finally, while animal reproductive studies have not been conducted with octreotide capsules (Mycapssa^®^), intravenous administration of octreotide in rats and rabbits at doses 7 and 13 times that of the suggested clinical dose has shown no adverse developmental effects in the offspring. While the safety and efficacy of the octreotide capsules (Mycapssa^®^) in the pediatric population has not been reported, the FDA-approved prescribing information suggests that post-marketing reports have noted risks of serious adverse reactions reported with octreotide injection use in pediatric patients, including hypoxia, necrotizing enterocolitis, and death. The safety and efficacy of the octreotide capsules (Mycapssa^®^) for treating gigantism in the pediatric population have not yet been established [17].

## 5. Conclusions

Acromegaly is a disorder of the anterior pituitary that results in the overproduction of GH by the anterior pituitary gland, often caused by a pituitary adenoma. The elevated GH levels subsequently cause an elevation in IGF-1 levels, which work synergistically to cause dysfunctions of both metabolic and physical development characterized by excessive growth of tissues. While women account for roughly half of the disease burden in acromegaly, female gender is disproportionately associated with delayed diagnosis and increased incidence of disease complications, including hypertension and diabetes, when compared to their male counterparts. Risks of acromegaly with uncontrolled GH and IGF-1 levels include tumor mass effect, cardiovascular complications, and respiratory complications, all contributing to an elevated mortality rate in these patients. Management of GH and IGF-1 levels is primarily accomplished through surgery or medical therapy. The first line of medical therapy is the somatostatin receptor analog drug class, including the somatostatin receptor ligand octreotide. Typically, octreotide administration is accomplished through monthly intramuscular injections or 3–4 daily subcutaneous injections, sometimes causing injection site pain, nodules, bruising, inflammation, and scarring. Most patients cannot self-administer these injections and, therefore, must visit their healthcare provider monthly for these injections.

Mycapssa^®^, the first FDA-approved oral octreotide capsule, provides a solution to the strain on patients and their healthcare team in medication therapy for the long-term management of acromegaly. Octreotide capsules (Mycapssa^®^) are a twice-daily oral administration capsule with clinical efficacy in maintaining IGF-1 and GH secretion at levels equivalent to those achieved by patients taking injected somatostatin receptor ligands. Using transient permeability enhancer (TPE) technology, octreotide capsules (Mycapssa^®^) combine octreotide with excipients, facilitating paracellular transport across the gastrointestinal wall into the small intestine. Across multiple clinical trials and long-term treatment studies, octreotide capsules (Mycapssa^®^) have maintained a consistent biochemical response and safety profiles similar to injected somatostatin receptor ligands and demonstrated a patient preference for continued treatment with oral octreotide capsules. While octreotide capsule (Mycapssa^®^) use is supported in women, further research is needed to assess the suggested risk of lactational transfer as well as drug tolerability at higher doses, as seen with the increased disease burden in women. Likewise, further research is needed to establish octreotide capsules (Mycapssa^®^) tolerability and efficacy in the pediatric population. Given these data, octreotide capsules (Mycapssa^®^) pose a possible solution for at-home medical therapy management of GH and IGF-1 levels in patients with acromegaly.

While oral octreotide capsules (Mycapssa^®^) have revolutionized the medical treatment of acromegaly by decreasing the disease management burden on the provider and patient alike, the drug’s TPE technology provides exciting opportunities for further enhanced bioavailability for oral administration of drugs previously restricted to subcutaneous injections. Future research should aim to assess oral octreotide capsule (Mycapssa^®^) tolerability at increased dosage as well as within specific populations like women and children. With the increased availability and accessibility of new treatment options, including oral octreotide capsules (Mycapssa^®^) for GH and IGF-1 management in patients with acromegaly, it is imperative that clinicians continue to improve diagnostic measures to seek earlier diagnosis, rapid onset of treatment, and earlier hormone suppression in acromegaly patients, especially females. Given an identical disease burden to their male counterparts, the diagnostic delay in women must be addressed with earlier screening and increased primary care outreach. With earlier disease diagnosis and minimally invasive treatment options like oral octreotide capsules (Mycapssa^®^), both patient burden and acromegaly disease management outcomes can continue to improve.

## Figures and Tables

**Table 1:** Summary of pharmacological approaches to acromegaly disease management.

Drug Class	Mechanism of Action	Indications for Use	Citation
Somatostatin Analogs	Directly binds to somatostatin receptors in both the normal pituitary and pituitary adenoma cells to suppress GH secretion.	First-line pharmacotherapy for acromegaly. Suppresses GH secretion in both normal pituitary and adenoma. Also suppresses IGF-1 secretion in the liver.	[4]
Dopamine Receptor Agonists	Directly binds to the pituitary D2R receptors to suppress GH secretion.	Serves as an adjunctive therapy, less effective than somatostatin analogs at GH and IGF-1 management.	[4]
GH- Receptor Agonists	Lowers IGF-1 levels via competitive inhibition of endogenous GH binding to GH receptors. No increased adverse events were associated with long-term therapy.	Adjunctive therapy, unable to suppress GH secretion but lowers IGF-1 by competitive inhibition of endogenous GH binding to hepatocyte receptors.	[4]

**Table 2:** Clinical trials of oral octreotide capsule efficacy and safety.

Groups Studied and Intervention	Results and Findings	Conclusions	Citation
Mycapssa^®^ CH-ACM-01: 155 patients with active acromegaly demonstrating biochemical control with injectable somatostatin receptor ligands were randomly assigned to be treated with octreotide capsules (Mycapssa^®^) twice a day with increasing dosages (40 mg/day, 60 mg/day, 80 mg/day) in there was inadequate suppression of IGF-1 for 13 months.	At the end of the 7-month core treatment period consisting of variable dosages of octreotide capsules (Mycapssa^®^), 65% of patients maintained normalization of GH and IGF-1 levels comparable to those of injected somatostatin receptor ligands. Of the 110 patients who entered the subsequent 6-month fixed-dose period, 80% maintained or improved acromegaly symptoms from baseline.	Oral octreotide capsules effectively maintain IGF-1 and GH level normalization in patients after switching from parenteral somatostatin receptor ligands to oral octreotide capsules for up to 13 months.	[6]
Mycapssa^®^ CHIASMA OPTIMAL: 56 patients with active acromegaly demonstrating biochemical control with injectable somatostatin receptor ligands were randomly assigned to groups to be treated with octreotide capsules (Mycapssa^®^) 40 mg/day, 60 mg/day, 80 mg/day or matching placebo capsules.	At the 36-week endpoint, 58.2% of the group treated with octreotide capsules (Mycapssa^®^) achieved normalization of IGF-1 levels, while only 19.4% of those treated with a placebo maintained IGF-1 measurements within their normal limits. GH levels were maintained in 77.7% of the octreotide capsules (Mycapssa^®^) treatment group and 30.4% of the placebo group.	Oral octreotide capsules are an effective therapy for long-term acromegaly maintenance (36 weeks) in patients previously treated with injectable somatostatin receptor ligands octreotide and lanreotide.	[5]
Mycapssa^®^ CHIASMA OPTIMAL OLE: 40 patients from the original OPTIMAL trial entered the open-label extension and were treated with 60 mg/day of the octreotide capsules (Mycapssa^®^) for 48 weeks.	At the 48th week of the open-label extension (OLE), 92.6% of the patients who responded to octreotide capsules (Mycapssa^®^) during the OPTIMAL trial maintained an equivalent normalization of IGF-1 levels throughout the OLE. Octreotide capsules’ (Mycapssa^®^) safety was consistent with prior findings, and no increased adverse events were associated with long-term therapy.	Oral octreotide capsules maintain efficacy in long-term treatment (48 weeks) of acromegaly patients with no indication for adverse events arising with long-term usage.	[25]
MPOWERED: Patients with a prior diagnosis of acromegaly under biochemical control entered a 36-week randomized controlled treatment with either oral octreotide capsules or injected somatostatin receptor ligands to monitor for adverse events and compare safety profiles.	During the 36-week randomized controlled treatment period, 19 patients in the oral octreotide capsule group and 15 in the injected somatostatin receptor ligand group had treatment-related adverse events. A total of 5.5% of the oral octreotide capsule group and 8.1% of the injected somatostatin receptor ligand reported serious adverse events.	Safety profiles of oral octreotide capsules are consistent with that of injectable somatostatin receptor ligands in treating acromegaly. No new risks were identified in the group treated with oral octreotide capsules.	[26]
The study assessed the absorption and effects on GH secretion in 75 healthy volunteers given oral octreotide doses of 3, 10, or 20 mg compared to 100 μg subcutaneous octreotide injection.	Both oral and subcutaneous injections were well tolerated, with escalating octreotide doses resulting in a dose-dependent increased octreotide concentration in plasma within 1 h after administration. Both 20 mg of oral octreotide and 0.1 mg of subcutaneously injected octreotide resulted in equivalent peak plasma concentrations and rate of plasma decay. The 20 mg dose of oral octreotide suppressed basal GH levels by 49% and GHRH-stimulated GH levels by 80%.	The study’s results support oral octreotide as an effective alternative to parenteral octreotide treatment in acromegaly due to demonstrating equivalent pharmacokinetic parameters with proper dose adjustment.	[21]

## Data Availability

Data are available upon request.
